# Regulatory Science for Veterinary Therapeutic Innovation: A Comparative Analysis of Drug Development Incentives Within the One Health Framework

**DOI:** 10.3390/vetsci13101119

**Published:** 2026-10-20

**Authors:** Sungwoo Hong, Sang-Myeong Lee

**Affiliations:** 1 College of Veterinary Medicine, Chungbuk National University, Cheongju-si 28644, Republic of Korea; sw.hong@rznomics.com; 2 R&D Center, Rznomics, Inc., Seongnam-si 13486, Republic of Korea

**Keywords:** veterinary drug development, regulatory science, one health, market failure, innovation policy, antimicrobial resistance, FDA-CVM, EMA, advanced therapeutics

## Abstract

Rapid advances in antiviral therapeutics, targeted biologics, RNA medicines, and precision veterinary medicine are generating unprecedented scientific opportunities for veterinary healthcare, yet translation of these innovations into approved veterinary products remains disproportionately slow. This review employs a comparative regulatory science framework to identify the regulatory and economic determinants of this innovation gap across four jurisdictions—FDA-CVM (US), EMA-CVMP (EU), PMDA/MAFF (Japan), and APVMA (Australia)—and to compare existing incentive instruments and identify the regulatory gaps between them. Two illustrative case studies, GS-441524 for feline infectious peritonitis and canine osteosarcoma therapeutics, illustrate how the absence of breakthrough pathways, limitations in the practical translation of Minor Use/Minor Species (MUMS) eligibility, and unresolved intellectual property barriers can suppress development investment. The multi-jurisdictional analysis indicates that this innovation gap is evident across the jurisdictions examined and is consistent with differences in the availability and strength of regulatory incentives, though scientific and market factors likely also contribute. Policy reforms are proposed, including a Veterinary Breakthrough Drug Designation, a One Health Innovation Designation with enhanced exclusivity, adaptive regulatory pathways, and strengthened VICH harmonization. Additionally, the regulatory preparedness for advanced veterinary therapeutics—including gene therapies, mRNA-based vaccines, and cell-based products—is examined as a critical frontier. The analysis highlights the role of regulatory science in translating emerging veterinary therapeutics into clinical practice within the One Health framework.

## Simple Summary

Developing new medicines for animals often takes longer and succeeds less often than developing medicines for people, even when the same disease affects both. Part of the reason may be that the rules and incentives that government agencies use to encourage new drug development are generally weaker for animals than for people, although scientific, market, and commercial factors also play a role. This paper compares how the United States, the European Union, Japan, and Australia support the development of new animal medicines, and identifies specific gaps in these systems using two real examples: a life-saving antiviral drug for a fatal cat disease whose regulated, prescribable version took years to reach the US market, and a bone cancer that affects both dogs and children but currently has no dog-specific approved treatment. The paper proposes practical reforms, including a faster review pathway for promising animal medicines and stronger incentives for treatments that benefit both animal and human health. It also examines whether current rules are ready for newer technologies such as gene therapies and RNA-based medicines in veterinary care. Better-designed rules for approving animal medicines would improve animal welfare, help control diseases that spread from animals to people, and support the responsible use of antibiotics in food-producing animals worldwide.

## 1. Introduction

Veterinary medicine is entering a period of unprecedented scientific opportunity. Advances in antiviral therapeutics, mRNA-based vaccines, gene therapies, targeted biologics, and AI-driven drug discovery are generating a pipeline of potential veterinary innovations comparable in scientific sophistication to their human pharmaceutical counterparts. Yet the translation of these scientific advances into approved veterinary products remains disproportionately slow. While human pharmaceutical pipelines generate between 22 and 59 novel drug approvals annually at the US FDA’s Center for Drug Evaluation and Research (CDER), the Center for Veterinary Medicine (CVM) typically approves fewer than 10 new animal drugs per year [1,2]. Multiple factors are likely to contribute to this disparity [3].

This innovation gap carries consequences that extend far beyond the veterinary sector. Veterinary medicinal products (VMPs) serve three foundational roles within the One Health framework: safeguarding animal welfare and agricultural productivity; preventing and managing zoonotic disease spillovers (influenza, coronaviruses, brucellosis, and others); and reducing selective pressure for antimicrobial resistance (AMR) in food-producing animals, thereby preserving the efficacy of medically important antibiotics for human use [4,5]. When veterinary therapeutic innovation stalls, all three of these One Health objectives are compromised.

This disparity likely reflects a combination of regulatory, economic, scientific, market, and commercial factors; differences in regulatory incentive architecture are one potentially important contributor that this review examines in detail. The practical disadvantages of veterinary drug development are well-documented. First, the veterinary market is fragmented across dozens of target species with distinct pharmacokinetic profiles, efficacy requirements, and regulatory data packages, fundamentally limiting economies of scale. Second, pricing flexibility is constrained by market dynamics, preventing risk-adjusted returns comparable to human therapeutics. Third, regulatory incentive architectures in the veterinary space lack the depth and breadth of their human counterparts, reducing the expected net present value (NPV) of veterinary R&D investment. These factors interact to create a valley of death in veterinary drug development that no single market actor can bridge unilaterally.

**Figure 1:**
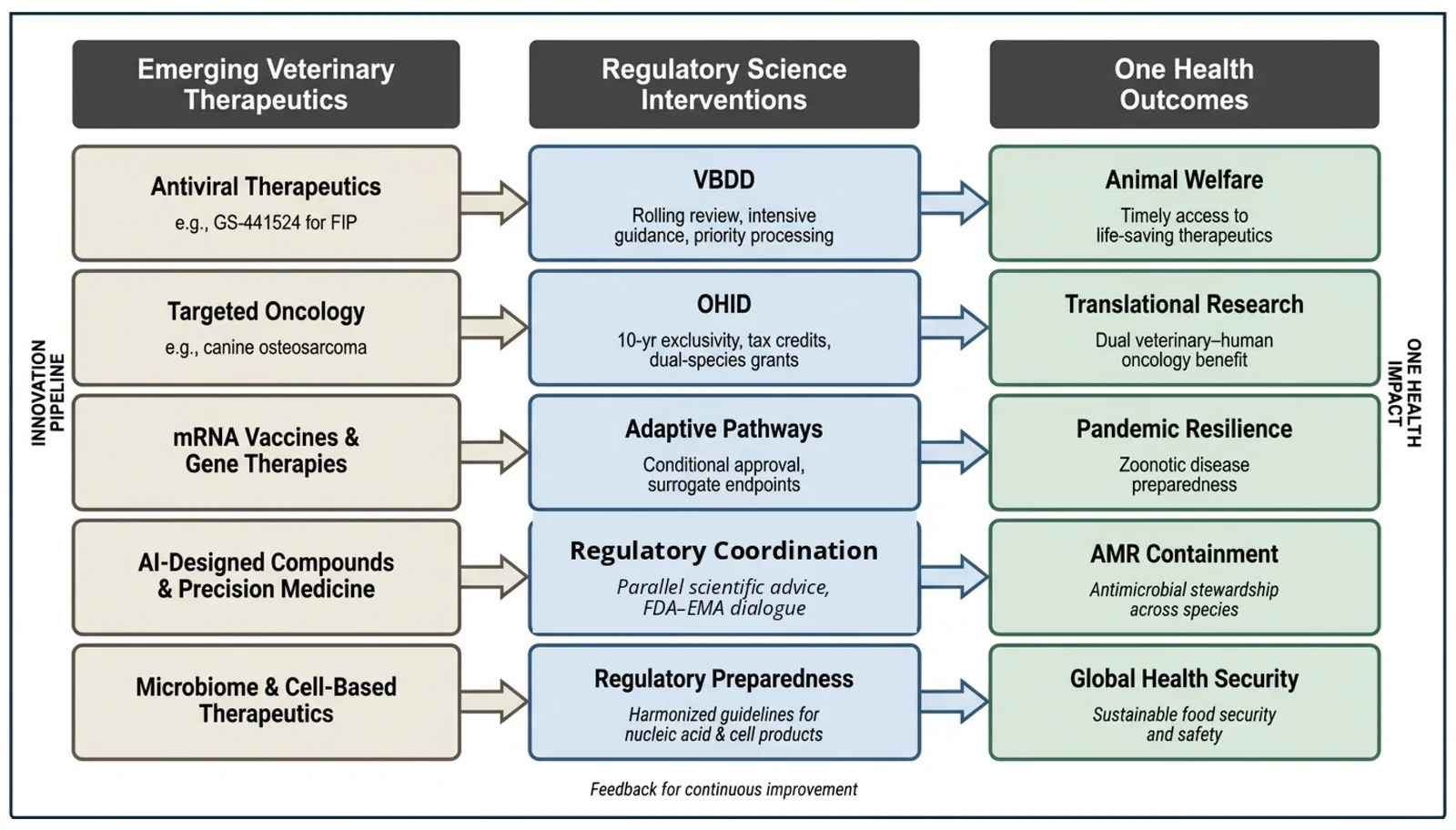
Regulatory science as an enabling discipline for veterinary therapeutic innovation within the One Health framework. This schematic illustrates how each category of emerging veterinary therapeutic (**left**) maps to the specific regulatory science interventions proposed in this review (**center**), which in turn generate measurable One Health outcomes (**right**). VBDD: Veterinary Breakthrough Drug Designation; OHID: One Health Innovation Designation. [Revised in this round: the “Regulatory Science Interventions” panel previously labeled “VICH Harmonization” (“Parallel scientific advice, PZAD expansion”) has been relabeled “Regulatory Coordination” (“Parallel scientific advice, FDA–EMA dialogue”) for consistency with the revised Section 4.2.4 and Table 3; the corresponding abbreviation for PZAD was removed from this caption as it no longer appears in the figure.].

This review employs a comparative regulatory science methodology to dissect the regulatory determinants of this innovation deficit. It systematically compares incentive architectures across US FDA-CVM and EMA-CVMP frameworks, extends the analysis to Japan (PMDA/MAFF) and Australia (APVMA), analyzes illustrative case studies of veterinary therapeutic translation, examines instances where regulatory pathways did not translate scientific eligibility into an approved product, and proposes policy options relevant to veterinary innovation and One Health. The analysis further considers the regulatory preparedness required to accommodate advanced veterinary therapeutics—gene therapies, RNA medicines, cell-based products, and AI-designed therapeutics—that are poised to transform veterinary medicine in the coming decade. Unlike conventional policy reviews, this article approaches regulatory science as an enabling scientific discipline—one that bridges emerging veterinary therapeutics with clinical translation and positions regulatory innovation as a prerequisite for therapeutic innovation, not simply a downstream consequence of it (Figure 1).

## 2. Methodology: A Comparative Regulatory Science Framework

This analysis employs a comparative regulatory science framework that evaluates the design, implementation, and outcomes of regulatory instruments across jurisdictions to compare differences in regulatory design and examine their policy implications. This is a narrative policy review, not a systematic literature review; accordingly, the methodology integrates three analytical dimensions without a formal systematic search protocol.

Regulatory architecture mapping: The incentive structures of FDA-CDER (human pharmaceuticals), FDA-CVM (US veterinary), and EMA-CVMP (EU veterinary) were mapped against six dimensions: (i) special designation programs; (ii) expedited review pathways; (iii) market exclusivity provisions; (iv) fee reduction and grant mechanisms; (v) adaptive approval frameworks; and (vi) international harmonization mechanisms via VICH. This mapping was extended to PMDA/MAFF (Japan) and APVMA (Australia) for comparative analysis.

**Figure 2:**
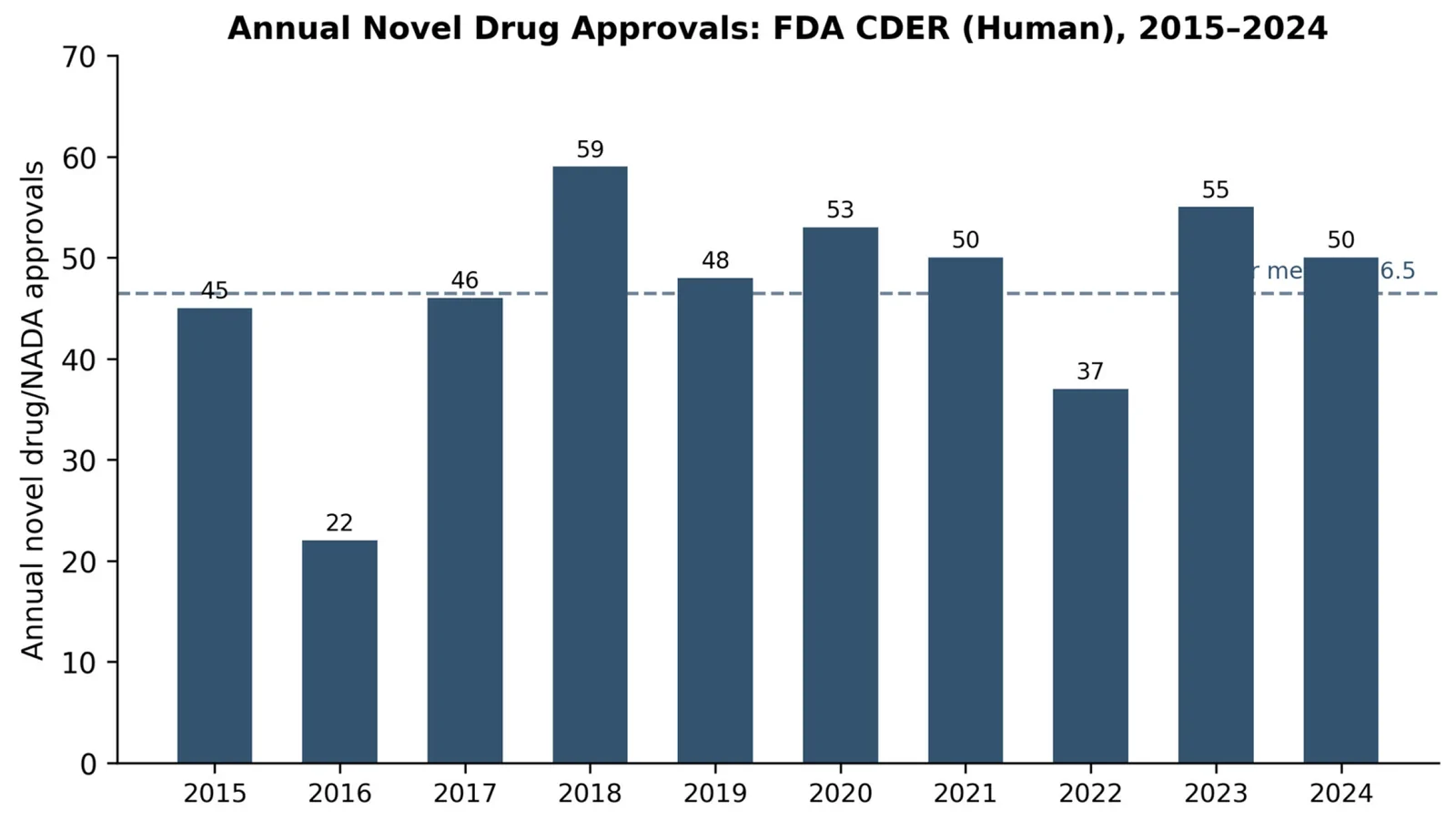
Annual Novel Drug Approvals: FDA CDER (Human), 2015–2024. Data from FDA Novel Drug Approvals annual reports [1]. A corresponding FDA CVM series was not included because the authors could not independently reproduce a verified annual count of novel NADA approvals excluding generic ANADAs, supplemental applications, and combination products from public Green Book records; see Section 3.2 and Limitations. The dashed line indicates the 10-year mean.

Quantitative benchmarking: Annual novel drug approval volumes for the human sector were obtained from FDA CDER Novel Drug Approvals annual reports (2015–2024) [1]. A corresponding veterinary-sector annual series from the FDA CVM Green Book was initially compiled by manual review but could not be independently reproduced with confidence in the inclusion/exclusion criteria applied (novel molecular entity approvals only, excluding generic ANADAs, supplemental applications, and combination products); this series is therefore not reported quantitatively, and Table 2 and Figure 2 present only qualitative and CDER-sourced indicators for the veterinary sector. Remaining indicators in Table 2 (pipeline depth, private R&D investment signal, user fee revenue) reflect a qualitative, literature- and publicly reported budget-document-based characterization rather than a systematic quantitative analysis of IND/INAD filings or R&D expenditure, and should be interpreted accordingly.

Case study analysis: Two cases—GS-441524 for feline infectious peritonitis (FIP) and therapeutic development for canine osteosarcoma—were purposively selected as illustrative, rather than representative, cases, based on three criteria: (i) high clinical unmet need, (ii) clear human therapeutic analogs enabling direct incentive architecture comparison, and (iii) documented regulatory divergence from human counterparts. These two cases were chosen to illustrate distinct mechanisms: the interaction of regulatory pathway limitations, sponsor participation, and intellectual-property constraints in Case 1, and the gap between formal MUMS eligibility and therapeutic translation in Case 2. They were not intended to represent the full population of veterinary drug development programs.

Limitations: This analysis relies on publicly available regulatory data, which may underrepresent the full range of failed or stalled development programs. A quantitative annual series of FDA CVM novel NADA approvals, initially compiled by manual review of Green Book records, could not be independently reproduced with confidence and has accordingly been omitted from Figure 2 and reported only qualitatively in Table 2; future work should establish a fully documented, reproducible extraction protocol for CVM approval counts. Economic modeling of NPV was qualitative-directional rather than quantitative, given the absence of comprehensive private investment data for the veterinary sector. The jurisdictional scope is limited to four regulatory systems (US, EU, Japan, Australia); emerging regulatory frameworks in East Asia (Republic of Korea, China) represent important comparators that warrant future dedicated analysis.

## 3. Comparative Regulatory and Economic Analysis

### 3.1. The Incentive Gap: Human vs. Veterinary Regulatory Frameworks

One important difference between human and veterinary drug development is the range of regulatory incentives available to reduce development risk and improve expected returns on R&D investment. Human pharmaceutical frameworks, particularly the US system pioneered by the Hatch–Waxman Act (1984) and the Orphan Drug Act (1983), deploy several mechanisms intended to encourage development in market segments where commercial incentives alone are insufficient. Veterinary frameworks have historically lagged in adopting equivalent instruments [3,6].

Table 1 provides a structured comparison of the key incentive categories across US human (CDER) and veterinary (CVM) frameworks, augmented with EU-EMA comparators.

**Table 1:** Regulatory Incentive Architecture: US Human (FDA-CDER) vs. US Veterinary (FDA-CVM). EU, Japanese, and Australian comparators are presented separately in Table 4.

Incentive Category	Human Pharmaceuticals (FDA-CDER)	Veterinary Medicinal Products (FDA-CVM)
Special Designations	ODD: 7-yr exclusivity, 25% tax credits (post-2018 rate), R&D grants, fee waivers. Breakthrough Therapy: intensive FDA guidance, rolling review. Fast Track, Priority Review, Accelerated Approval.	MUMS Act (2004): 7-yr exclusivity, minor species and minor uses in major species. No broad Breakthrough equivalent. CNADA available but rarely used.
Expedited Pathways	Breakthrough Therapy, Fast Track, Priority Review, Accelerated Approval (surrogate endpoints). Rolling review standard.	No broad expedited pathway equivalent. Phased review allows sectional submission but not priority processing.
Market Exclusivity	NCE: 5-yr data exclusivity. ODD: 7-yr market exclusivity. Biologics: 12-yr. Patent term restoration available.	NCE: 5-yr data exclusivity. MUMS: 7-yr (minor species and minor uses in major species). Patent term extension available under 35 U.S.C. §156 for qualifying new animal drugs (e.g., CYDECTIN, IMPROVEST, ZILMAX).
Fee Waivers & Grants	ODA grants: up to USD 700 K/yr. SBIR/STTR grants. Orphan Product Development grants. PDUFA fee waivers for orphan designations.	MUMS grants: available but smaller. ADUFA/AGDUFA user fees apply; limited waivers. No equivalent to SBIR/STTR for veterinary drug R&D.
International Harmonization	ICH: highly developed framework across US, EU, Japan, Canada.	VICH: operational but slower pace.

Note: CDER and CBER share major expedited review mechanisms including Fast Track, Breakthrough Therapy, and Priority Review designations. CBER additionally operates the Regenerative Medicine Advanced Therapy (RMAT) designation, which applies specifically to cell and gene therapy products. For consistency in quantitative comparison, this study used CDER data as the reference standard. MUMS: Minor Use and Minor Species; NCE: New Chemical Entity; ODA: Orphan Drug Act; ODD: Orphan Drug Designation; ADUFA: Animal Drug User Fee Act.

### 3.2. Quantitative Benchmarks of the Innovation Deficit

FDA CDER approved between 22 and 59 novel drugs annually during 2015–2024, with an average of approximately 47 approvals per year [5]. Direct quantitative comparison with FDA CVM is difficult because publicly available veterinary approval records include multiple application types, and the annual series initially compiled for this review could not be independently reproduced using a directly comparable definition of novel drug approval. Accordingly, the CDER data are presented descriptively and are not interpreted as quantitative evidence that differences in regulatory incentives cause differences in innovation output. The available descriptive indicators are summarized in Table 2 and Figure 2.

**Table 2:** Innovation Indicators: Human vs. Veterinary Sectors (2015–2024), Combining Quantitative Data and Qualitative Characterization.

Indicator	Human Medicine (CDER)	Veterinary Medicine (CVM)
Annual novel drug approvals (NDA/NADA)	22–59 (avg. ~47)	Substantially lower (exact annual counts not independently reproducible; see Limitations)
Clinical/field trial intensity	High (thousands of trials)	Low and fragmented
Pipeline depth (INDs filed/year)	Strong and predictive	Weak and reactive
Private R&D investment signal	Self-reinforcing cycle	NPV-negative for many species
User fee revenue	PDUFA: ~USD 1.5 B/yr	ADUFA: ~USD 30 M/yr

NDA: New Drug Application; NADA: New Animal Drug Application; IND: Investigational New Drug; INAD: Investigational New Animal Drug. PDUFA/ADUFA fee estimates from FDA budget documents (FY2024).

### 3.3. Illustrative Case Studies in Veterinary Therapeutic Translation

Both case studies involve companion-animal diseases that are not zoonotic in the conventional sense. Their relevance to the One Health framework therefore lies in the animal-welfare and comparative-medicine roles identified in the Introduction, not in zoonotic transmission. The GS-441524/FIP case centers on the animal-welfare and veterinary therapeutic-access dimension: the consequences for cats of a prolonged absence of a formally approved treatment pathway. The canine osteosarcoma case reflects the comparative-medicine and translational-oncology dimension, given the disease’s histological and molecular similarity to pediatric human osteosarcoma (Section 3.3.2). Zoonotic disease transmission and antimicrobial resistance are addressed separately, as additional One Health dimensions, in Section 4.3.

#### 3.3.1. GS-441524 and Feline Infectious Peritonitis: The Cost of Absent Breakthrough Pathways

Feline infectious peritonitis (FIP), caused by a mutant feline coronavirus (FCoV), was historically a near-universally fatal disease in cats, with case fatality rates approaching 100% in untreated animals. Two classes of antiviral agents demonstrated remarkable clinical efficacy against FIP in pioneering studies published from 2018 onward: the 3C-like protease inhibitor GC376, tested in a cohort of 20 cats with various forms of FIP [7]; and the nucleoside analog GS-441524, a metabolic precursor of the active triphosphate form of remdesivir (GS-5734), which demonstrated high efficacy and safety in a subsequent clinical study [8]. GS-441524 has since become widely used for FIP treatment, and a retrospective observational study of 629 treated cats reported an overall treatment success rate of approximately 85% [9].

The regulatory trajectory of GS-441524 for FIP illustrates how regulatory, intellectual-property, and market factors can interact in veterinary drug development. Remdesivir, which is structurally related to GS-441524, was developed within the human-drug regulatory system, whereas no veterinary counterpart to the human Breakthrough Therapy or Fast Track designations was available for GS-441524. In 2018, Congress expanded FDA-CVM’s statutory authority for conditional approval to include certain major uses in major species; FDA subsequently finalized Guidance for Industry #261 in July 2021 to clarify eligibility criteria for this expanded pathway. GS-441524 did not enter a formal FDA veterinary development pathway, and sponsor and intellectual-property constraints also limited the prospect of conventional sponsor-led development [10].

However, the regulatory gap alone does not fully explain the delay of more than five years between the 2018–2019 publication of clinical efficacy data for GS-441524 and FDA’s May 2024 enforcement discretion policy permitting compounded access. Three distinct barriers operated simultaneously during this interval:

(i) Regulatory pathway absence: No veterinary breakthrough designation existed at FDA-CVM that could provide intensive guidance, rolling review, or priority processing for a compound treating a uniformly fatal disease. This contrasts with the human Breakthrough Therapy Designation available since 2012.

(ii) Intellectual property barrier: According to a contemporaneous legal-commentary account, the patent holder did not license GS-441524 for veterinary development [10]; we were not able to independently verify this through primary documentation such as licensing records or company statements, and note it as a plausible but not conclusively documented contributing factor. If accurate, it would illustrate a general limitation of the current framework: a breakthrough designation cannot compel a patent holder to sponsor development, and no mechanism exists at FDA-CVM to authorize third-party development when a patent holder declines to pursue veterinary applications of a promising compound.

(iii) Compounding as de facto alternative pathway: In the United States, the practical consequence was that the only route to treatment access for FIP patients was through unregulated procurement of GS-441524, primarily from unlicensed overseas sources, for a period exceeding five years [10,11]. Notably, other jurisdictions—including Australia, the United Kingdom, Canada, and the Netherlands—established earlier access through compounding pharmacy frameworks and off-label use of remdesivir, creating an international regulatory asymmetry [9,12].

The absence of a formal regulatory framework in the US created patient safety risks from unverified product quality, precluded systematic pharmacovigilance data collection, and denied the clinical community the ability to conduct properly supervised field trials. On 10 May 2024, the FDA clarified its enforcement discretion policy, indicating that it does not intend to enforce new animal drug approval requirements for products compounded from GS-441524 when prescribed by a veterinarian for FIP treatment under Guidance for Industry #256 [13]. It is important to note that this is not a conditional approval or authorization; compounded GS-441524 remains an unapproved drug, and the FDA explicitly reminded stakeholders of this status [13]. This enforcement discretion pathway bypasses formal drug development entirely.

This case illustrates that addressing the veterinary innovation deficit requires interventions across all three layers simultaneously. A veterinary breakthrough pathway would have enabled structured regulatory engagement, but its impact would have been limited without parallel mechanisms to address IP barriers, such as provisions for third-party development authorization or march-in-like instruments when patent holders decline to pursue development for uniformly fatal veterinary diseases. The international comparison further demonstrates that the regulatory design choices of individual jurisdictions have direct, measurable consequences for patient access timelines.

#### 3.3.2. Canine Osteosarcoma: Regulatory Eligibility Does Not Guarantee Therapeutic Translation

Canine osteosarcoma is the most common primary bone tumor in dogs, affecting an estimated 8000–10,000 dogs annually in the United States [14]. Histologically and molecularly, it shares significant features with pediatric osteosarcoma in humans, making the canine model one of the most clinically relevant for translational oncology research. Despite this high unmet need and scientific importance, the therapeutic pipeline for canine osteosarcoma is notably sparse [3].

Canine osteosarcoma is not categorically excluded from the MUMS framework because dogs are classified as a major species: the MUMS Act also covers minor uses in major species, and FDA specifically identifies control of pain associated with bone cancer in dogs as an example of a qualifying minor use. FDA’s Designations List includes two products granted MUMS designation for control of pain associated with canine bone cancer—resiniferatoxin (designated 19 June 2015; designation terminated 3 December 2024) and SP-SAP (designated 9 July 2015; designation terminated 28 March 2019) [15]. These designations addressed bone-cancer-associated pain, not osteosarcoma itself as an antineoplastic indication, but they demonstrate that the MUMS pathway has in fact been accessible for this disease area.

The MUMS framework has also facilitated successful oncology drug development in dogs more broadly. FDA regulatory impact data covering August 2009 through April 2021 identify 20 MUMS designations for minor uses in dogs, most involving neoplastic conditions such as leukemia, melanoma, sarcoma, and lymphoma [16]. Rabacfosadine (Tanovea) and verdinexor (Laverdia), both developed for canine lymphoma, progressed from MUMS conditional approval to full approval in July 2021 and December 2025, respectively [15,17].

Nevertheless, FDA’s current list of approved canine cancer therapies—toceranib (Palladia) and tigilanol tiglate (Stelfonta) for mast cell tumor, and rabacfosadine and verdinexor for lymphoma—includes no product specifically indicated for osteosarcoma [18], and both bone-cancer-pain MUMS designations noted above were subsequently terminated. These contrasting outcomes do not establish that the regulatory framework itself caused the limited osteosarcoma pipeline; rather, they illustrate that formal MUMS eligibility does not by itself determine development success, which may also depend on disease biology, clinical-trial feasibility, sponsor interest, market size, development costs, and expected commercial return.

This case therefore does not motivate a One Health Innovation Designation on the grounds that canine osteosarcoma is excluded from existing MUMS mechanisms. Rather, it illustrates a gap in recognition: MUMS is designed to offset the limited commercial return of a small veterinary indication, but it does not specifically reward the additional translational value that a canine osteosarcoma development program could generate for pediatric osteosarcoma research in humans, given the substantial histological and molecular similarity between the two diseases. A One Health Innovation Designation—distinct from MUMS and Orphan Drug Designation—could recognize this dual-use translational value explicitly, providing an incentive layer oriented toward the human-relevance dimension of veterinary development, instead of substituting for MUMS itself.

### 3.4. VICH: The Harmonization Engine and Its Limits

The International Cooperation on Harmonisation of Technical Requirements for Registration of Veterinary Medicinal Products (VICH) was officially launched in April 1996 as the veterinary counterpart to the human ICH framework. Its Steering Committee unites regulatory authorities (FDA-CVM, USDA-APHIS, EMA, EU Commission, MAFF of Japan) and industry representatives, establishing a trilateral US-EU-Japan harmonization architecture [19].

VICH’s substantive contribution to veterinary regulatory infrastructure has been considerable. Since 1996, VICH has adopted over 60 guidelines (GL1–GL61 as of this writing) spanning analytical validation, safety pharmacology, toxicology, pharmacovigilance, quality, and efficacy domains [19,20]. The most recent additions include VICH GL60 (GMP for APIs, adopted 2024), VICH GL61 (Pharmaceutical Development, finalized at Step 7 in April 2026), and VICH GL23 Revision 2 (Genotoxicity Testing, finalized at Step 7 in August 2025), the last of which formally introduced QSAR-based computational predictions as acceptable supplementary data, representing the first explicit VICH incorporation of an in silico new approach methodology [20].

Despite these achievements, VICH’s institutional design generates three systematic limitations that reduce its effectiveness as an innovation catalyst.

Limitation 1: Differences in mandate and scope relative to ICH. ICH and VICH differ substantially in mandate, scope, product coverage, membership, and guideline structure. Accordingly, the number of guidelines issued by each organization should not be interpreted as a comparative measure of regulatory effectiveness, institutional capacity, or harmonization performance. The relevant policy question is instead whether veterinary-specific technical guidance keeps pace with emerging product classes and development methods within VICH’s mandate.

Limitation 2: The Standing Member gap. VICH operates a three-tier participation structure: Founding Members (EU, US, Japan), who hold voting rights on the Steering Committee; Standing Members without voting rights (Australia, New Zealand, Canada, South Africa, and the United Kingdom), who may participate in Committee discussions and Expert Working Groups; and Observers (Switzerland, represented by Swissmedic and Scienceindustries), who are not bound by VICH recommendations but are encouraged to take them into account, alongside WOAH as an Associate Member.

Limitation 3: Innovation incentives outside scope. VICH guidelines address technical data requirements but not regulatory incentive instruments. Designation programs, expedited review pathways, market exclusivity provisions, and tax incentive frameworks are entirely outside VICH’s mandate. A manufacturer developing a veterinary antimicrobial for all three major markets must navigate three substantially different incentive architectures simultaneously, without harmonized guidance [19].

**Table 3:** VICH Guideline Development: Key Domains and Innovation Relevance.

GL Range	Domain	Key Content/Latest Update	Innovation Relevance
GL1–GL16	Analytical & Quality	Validation, stability, impurities, GMP for APIs (GL60, 2024)	Reduces regulatory uncertainty; lowers compliance cost
GL22–GL33	Safety Toxicology	Residue safety, reproduction, genotoxicity (GL23 R2, finalized August 2025, incl. QSAR)	Reduces duplicative testing; first NAMs integration
GL36–GL45	Pharmacovigilance	Adverse event management, periodic safety updates, signal detection	Harmonizes post-market surveillance
GL46–GL61	Efficacy & Quality (new)	GCP (GL9 revised), pharmaceutical development (GL61, finalized April 2026)	Reduces clinical development variation; supports MRA-based submissions
Pending	Adaptive Pathways/NAMs	No VICH guideline exists; active gap as of 2024–2025	Outside current VICH scope; no cross-jurisdictional framework for adaptive/conditional approval or OoC data

GL: VICH Guideline; NAMs: New Approach Methodologies; OoC: Organ-on-a-Chip; GCP: Good Clinical Practice; MRA: Mutual Recognition Agreement.

### 3.5. International Comparators: PMDA (Japan) and APVMA (Australia)

#### 3.5.1. Japan: PMDA’s Dual-Track Problem in Veterinary Regulation

Japan’s Pharmaceuticals and Medical Devices Agency (PMDA) operates under the Pharmaceuticals and Medical Devices Act (PMD Act, 2014; substantially amended 2025). PMDA has developed a sophisticated expedited pathway architecture for human pharmaceuticals—including Sakigake designation targeting a 6-month review; Priority Review (9-month target); Conditional Early Approval; and Orphan Drug Designation with 20–40% fee reduction, tax credits, and premium pricing [21,22]. In FY2024, PMDA achieved the shortest median approval time among six major global regulatory authorities—290 days—compared to 356 days at FDA CDER [23].

However, this performance profile is specific to human pharmaceuticals. The veterinary pharmaceutical approval system operates through a separate framework under MAFF and its Pharmaceutical and Feed Safety Division, which lacks equivalents to Sakigake designation, Conditional Early Approval, or any formalized expedited pathway [21]. This dual-track problem—world-class human pharmaceutical regulation coexisting with a comparatively static veterinary regulatory framework—illustrates that regulatory authority sophistication does not automatically transfer across sectors even within a single jurisdiction. The 2025 PMD Act amendments, enacted by Japan’s Diet on 14 May 2025 to address medicine quality, supply, and drug-shortage issues [24], represent legislative momentum that could, with targeted advocacy, be extended to veterinary pharmaceuticals in future amendment cycles.

#### 3.5.2. Australia: APVMA’s Permit-Based System and the Minor Use Architecture

Australia’s veterinary pharmaceutical regulator, the Australian Pesticides and Veterinary Medicines Authority (APVMA), operates under the Agricultural and Veterinary Chemicals Code (Agvet Code, 1994) to centralize registration of all agricultural and veterinary chemical products [25]. APVMA operates a permit-based access system for minor use and minor species scenarios that provides a pragmatic alternative to formal registration [26]. This permit system serves a function analogous to the US MUMS designation but without conferring market exclusivity or development incentives; it functions as an access mechanism, not an innovation incentive.

APVMA is actively modernizing its technical infrastructure. The 2024 Veterinary Immunobiological Products guideline revision (draft 2025), updated minor use guidelines (August 2025), and exploration of mRNA veterinary vaccine regulatory frameworks reflect an agency in active regulatory evolution [27]. APVMA’s August 2024 Newsletter documented active engagement with mRNA veterinary vaccines—for which no APVMA-approved products yet exist—indicating a proactive horizon-scanning posture [28]. Australia’s position as a VICH Standing Member means APVMA may participate in VICH Committee discussions and Expert Working Groups but has no voting rights in guideline development.

**Table 4:** Multi-Jurisdictional Veterinary Regulatory Incentive Architecture.

Dimension	FDA-CVM (US)	EMA-CVMP (EU)	MAFF (Japan)	APVMA (Australia)
Legal Framework	FD&C Act; MUMS Act (2004); ADUFA (reauth. 2023)	Regulation (EU) 2019/6 (Jan 2022)	PMD Act (2014; rev. 2025); MAFF notifications	Agvet Code (1994; ongoing amendment)
Expedited Pathway	CNADA (conditional); no vet Breakthrough	VLM authorization; no vet PRIME	No vet-specific expedited pathway	No equivalent expedited pathway
Designation Incentives	MUMS 7-yr exclusivity (minor species and minor uses in major species); PZAD (2023)	Limited market data protection (10 yr)	No vet-specific designation incentive	No designation-based exclusivity
VICH Membership	Full member	Full member	Full member	Standing Member (non-voting)
Key Innovation Gap	No vet Breakthrough; MUMS eligibility does not guarantee translation	Fee increases may deter new VMPs	World-class human/static vet system	Permit = access, not incentive
Recent Reform	PZAD (2023); NAMs roadmap (2025)	EU 2019/6 VLM implementation	PMD Act 2025 amendments (human)	Immunobiologicals guideline (2025); mRNA VVx

VLM: Veterinary Limited Market; CNADA: Conditional New Animal Drug Application; PZAD: Priority Zoonotic Animal Drug; MAFF: Ministry of Agriculture, Forestry and Fisheries; VVx: veterinary vaccine.

### 3.6. Regulatory Timelines: Cross-Jurisdictional Milestone Analysis

**Figure 3:**
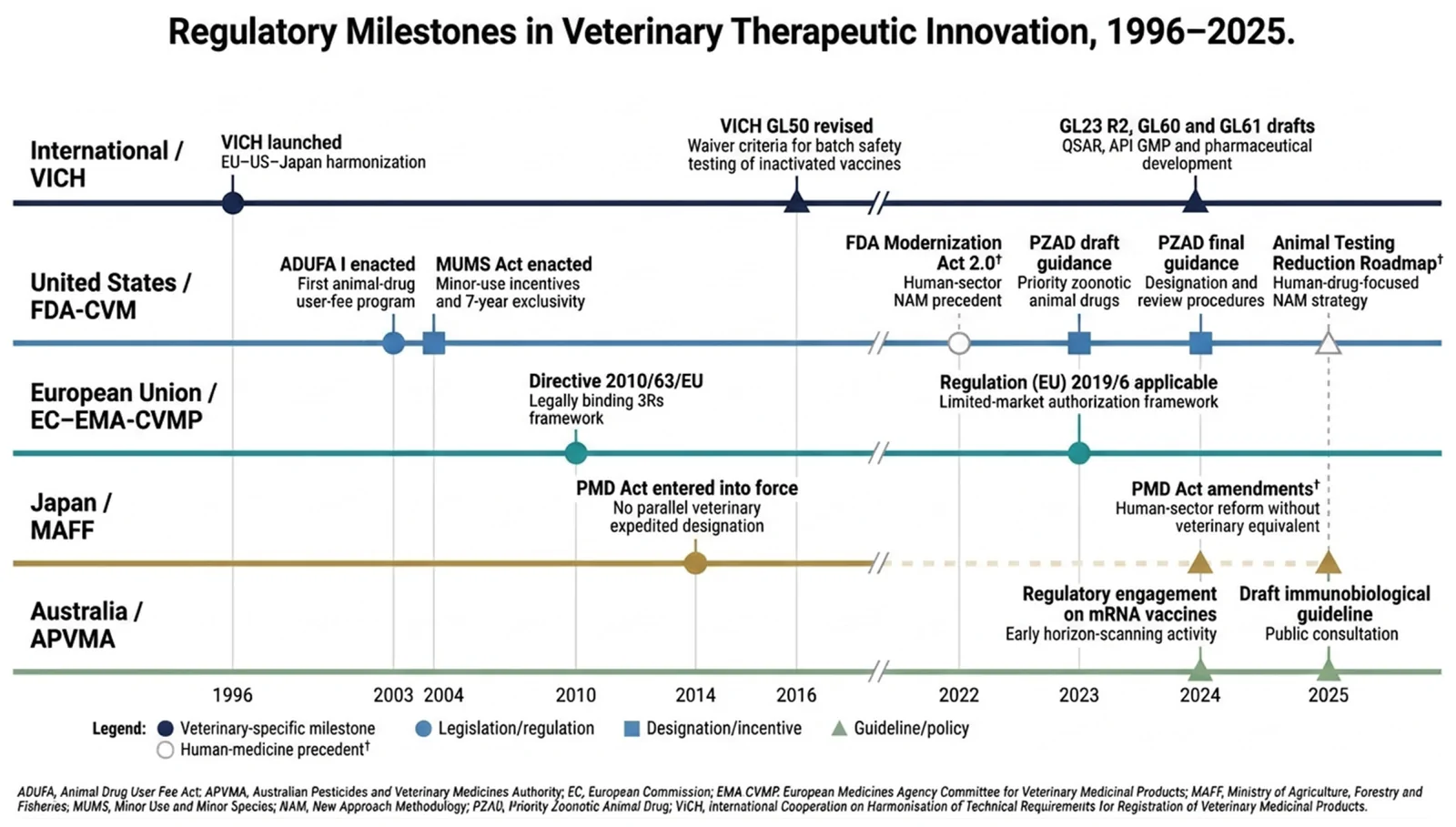
Regulatory milestones in veterinary therapeutic innovation across major jurisdictions, 1996–2025. The swimlane timeline presents selected regulatory and policy developments relevant to veterinary medicinal product innovation across VICH, the United States, the European Union, Japan, and Australia. Marker colors identify the corresponding jurisdiction, whereas marker shapes distinguish legislation or regulation, designation or incentive mechanisms, and technical guidelines or policies. Dagger symbols (†) identify human-medicine regulatory initiatives included as cross-sector precedents, not veterinary-specific legal changes. The dashed segment in the Japan/MAFF lane denotes the absence of a corresponding veterinary expedited pathway during the period shown, rather than discontinuity of the underlying regulatory framework. The broken time axis expands the 2022–2025 period, during which regulatory activity became increasingly concentrated.

Figure 3 maps selected regulatory and policy milestones across five swimlanes representing VICH, the United States, the European Union, Japan, and Australia. Veterinary-specific reforms are distinguished from human-sector regulatory initiatives included as cross-sector precedents.

Three patterns emerge from the timeline. First, veterinary-specific incentive reforms have occurred intermittently, not as a coordinated global progression. Second, the period from 2022 to 2025 shows an acceleration of regulatory activity, particularly in limited-market authorization, zoonotic-drug designation, technical harmonization, and new approach methodologies. Third, several prominent reforms in the United States and Japan remain confined to human medicinal products, underscoring the persistent failure of regulatory innovation to transfer systematically into the veterinary sector.

It is also important to note the jurisdictional fragmentation of veterinary regulatory authority within the United States. While FDA-CVM regulates veterinary drugs, biological products for veterinary use—including vaccines—fall under the jurisdiction of the USDA-APHIS Center for Veterinary Biologics (CVB). This split creates an additional regulatory barrier for innovative products that span the drug–biologic boundary, such as mRNA-based veterinary vaccines, therapeutic vaccines, and gene therapy products. No harmonized expedited pathway exists across the CVM-CVB divide, and designation incentives such as MUMS apply only to CVM-regulated products [3].

## 4. Discussion: Revitalizing the Veterinary Drug Innovation Ecosystem

### 4.1. The Self-Reinforcing Cycle and How to Break into It

In the human pharmaceutical sector, regulatory incentives and private investment interact within a self-reinforcing cycle. Robust regulatory instruments reduce development risk and enhance NPV expectations, which attract private capital, which funds clinical infrastructure that generates data, which enables further approvals. Veterinary drug development is effectively excluded from this cycle by the thinness of its incentive architecture.

The key intervention point is the expected NPV of veterinary R&D, which is a function of three variables: (i) the probability of regulatory success; (ii) the discounted value of future revenues, determined by market size, exclusivity period, and pricing flexibility; and (iii) the time to approval, which determines the fraction of patent protection remaining upon market entry. Current veterinary incentive frameworks negatively impact all three variables simultaneously. Reform would need to address several aspects of the development process at once: timeline compression (adaptive pathways), revenue protection (enhanced exclusivity), and regulatory certainty (pre-submission scientific advice and clear designation criteria).

### 4.2. Proposed Policy Reforms

#### 4.2.1. Veterinary Breakthrough Drug Designation Pathway

The FDA should establish a Veterinary Breakthrough Drug Designation (VBDD) for VMPs that (i) treat, prevent, or mitigate serious or life-threatening animal diseases for which no adequate approved therapy exists; or (ii) demonstrate a substantial improvement over available therapies based on preliminary clinical evidence. VBDD would confer: intensive FDA-CVM guidance beginning as early as the INAD stage; rolling review of the NADA; organizational commitment of senior FDA-CVM staff; and priority review designation at NADA filing.

For the GS-441524/FIP case, a VBDD-type mechanism could have provided earlier and more structured regulatory dialogue if an eligible sponsor had pursued formal development. The available evidence does not support estimating how many years such a pathway would have shortened development, and regulatory facilitation alone would not have resolved sponsor participation or intellectual-property constraints. The case therefore supports considering earlier regulatory engagement as one component of a broader development framework. It does not support assuming a specific counterfactual approval timeline.

#### 4.2.2. MUMS Designation Reform and One Health Innovation Designation

Two complementary reforms are proposed. First, FDA-CVM should establish a dedicated “Major Species Unmet Need” priority track within the existing MUMS framework, giving explicit visibility and procedural priority to minor-use development in dogs and cats for life-threatening conditions lacking an adequate approved therapy, addressing the gap between formal MUMS eligibility and realized therapeutic translation documented in Section 3.3.2, rather than creating a new eligibility category. Second, Congress should enact a One Health Innovation Designation (OHID) for VMPs that address diseases with significant zoonotic potential or where the veterinary program generates evidence directly informative for human drug development. OHID should confer: 10-year market exclusivity; transferable tax credits; and eligibility for One Health development grants funded jointly by NIH and USDA.

#### 4.2.3. Adaptive Regulatory Pathways for Veterinary Medicines

The conditional approval pathway (CNADA) provides a foundation for an adaptive regulatory framework but requires significant expansion. CVM should adopt an expedited-development framework analogous to EMA’s PRIME (PRIority MEdicines) scheme. PRIME is not itself an approval pathway and does not lower the evidentiary standard for marketing authorization; it is a support scheme that provides sponsors of medicines for an unmet human medical need with earlier and more intensive EMA scientific advice, an early-appointed rapporteur, and coordinated evidence-generation planning, helping them meet the standard evidentiary requirements more efficiently and, where eligible, supporting use of the EU’s existing accelerated-assessment route. A veterinary analogue could similarly emphasize early scientific advice, coordinated evidence planning, and prioritized review for high-unmet-need products, and could support conditional marketing authorization at FDA-CVM based on surrogate endpoints or limited field trials, with post-marketing confirmation studies required within a defined timeline. Under 21 CFR Part 514, a CNADA allows FDA-CVM to grant conditional approval based on a “reasonable expectation of effectiveness” rather than the full substantial evidence of effectiveness required for standard approval, provided the sponsor has demonstrated safety; conditional approval is valid for one year and renewable annually for up to five years while the sponsor completes the effectiveness studies needed for full approval. The Priority Zoonotic Animal Drug (PZAD) designation is a separate, more recent instrument: FDA finalized its PZAD guidance (GFI #283) in June 2024 to provide expedited review for animal drugs addressing zoonotic disease threats [29]; because the designation is still new, its real-world uptake and practical limitations are not yet established in the public record, and neither CNADA nor PZAD currently confers market exclusivity or tax incentives comparable to the human Orphan Drug or Breakthrough Therapy programs.

The EU’s Regulation (EU) 2019/6, which took effect in January 2022, introduced extended data protection for VMPs addressing limited market conditions [30,31]. However, as documented by Hellmann et al. in a 2026 Frontiers in Veterinary Science conference report, the provisions intended to foster innovation have been limited in practice to extension of data protection periods for limited market VMPs and the specification of requirements for certain novel product types such as cell therapies, while the fee structure has increased substantially, functioning as a disincentive for novel VMPs [32]. The present analysis extends beyond the EU-focused scope of Hellmann et al. by providing a four-jurisdiction comparative framework, quantifying the human–veterinary incentive disparity, and proposing specific designation instruments (VBDD, OHID) designed to address the regulatory gaps identified across all four regulatory systems.

#### 4.2.4. Enhanced International Harmonization: VICH and Bilateral Pathways

FDA and EMA already operate a Parallel Scientific Advice Program for new animal drugs (updated June 2024) [33]. One Health-designated products and VBDD-eligible candidates could be given priority access to this existing sponsor-facing advice mechanism; its scope and function would stay the same. Additionally, the Priority Zoonotic Animal Drug (PZAD) designation, introduced by FDA in 2023, provides a valuable template for internationally coordinated rapid review of zoonotic-relevant VMPs [29]. Because designation and expedited-review programs of this kind fall outside VICH’s technical harmonization mandate (Section 3.4), we propose PZAD-style coordination be pursued through a separate multilateral or bilateral mechanism, distinct from the Parallel Scientific Advice Program’s existing sponsor-facing function, instead of through VICH itself. In the near term, FDA and EMA could address incentive coordination through a separate regulator-to-regulator dialogue, distinct from the Parallel Scientific Advice program’s existing sponsor-facing function. The two agencies’ existing administrative cooperation could serve as a starting point, and no new treaty-level commitments would be required. A broader multilateral forum, either a reconstituted VICH mandate that explicitly covers incentive harmonization or a new body created for that purpose, could ultimately provide wider coverage across VICH Founding and Standing Members, but establishing its membership, mandate, and legal basis requires analysis beyond the scope of the present review. We mention it here as a longer-term possibility, not a near-term recommendation.

#### 4.2.5. Implementation Pathway, Funding, and Safeguards

The two proposed designations differ in the statutory authority they require. The Veterinary Breakthrough Drug Designation (VBDD, Section 4.2.1) and the Major Species Unmet Need priority track (Section 4.2.2) are procedural review-track mechanisms comparable to FDA’s existing Breakthrough Therapy and MUMS designations. These mechanisms include procedural features—such as earlier sponsor engagement, enhanced scientific advice, and prioritized internal coordination—that could plausibly be implemented administratively by FDA-CVM, and features that would create new legal entitlements or incentives and would more likely require additional statutory authority; we present administrative implementation as a plausible pathway that could be incorporated into the next ADUFA reauthorization cycle, rather than a definitive legal conclusion, and note this would still require additional FDA-CVM staff capacity for earlier-stage sponsor engagement, a resource question that would need to be negotiated as part of that reauthorization cycle; it should not be assumed to be cost-free. The One Health Innovation Designation (OHID, Section 4.2.2), by contrast, creates new market exclusivity and a transferable tax credit and would therefore require congressional action, analogous to the original Orphan Drug Act; funding for the associated NIH/USDA joint development grants would be subject to ordinary appropriations rather than a dedicated funding stream, and program-level costs are not estimated here. The proposed 10-year OHID exclusivity period would need to interact with other applicable exclusivities the way comparable veterinary exclusivities already do: FDA’s own regulatory-impact analysis of the MUMS Act states that the 7-year MUMS exclusive marketing period may overlap with some or all of the separate 5-year marketing exclusivity available to qualifying new animal drugs under GADPTRA; the two are not mutually exclusive alternatives [16]. We do not assume that a proposed 10-year OHID exclusivity period would automatically add to existing MUMS or standard new-animal-drug exclusivity for the same product. How it would interact with other applicable exclusivities, whether running concurrently, offsetting them, or limiting them in some other way, would need explicit statutory specification by Congress. At this stage, strict eligibility criteria are more important than specifying a particular stacking rule: the eligibility criteria in Section 4.2.2 (significant zoonotic potential or direct relevance to human drug development) and the reporting requirement described below are intended to limit OHID to products with additional One Health or translational value, not to let it function as a general-purpose route to a longer exclusivity period. A complete statutory design, including how FDA-CVM would adjudicate borderline eligibility claims, is beyond the scope of the present analysis.

To avoid inadvertently weakening antimicrobial stewardship, eligibility for OHID or VBDD status for any antimicrobial VMP should be conditioned on the sponsor’s submission of a stewardship and post-marketing antimicrobial-use monitoring plan consistent with FDA’s existing GFI #263 framework for veterinary oversight of medically important antimicrobials [34]; this safeguard is not intended as a complete design and would require further specification by FDA-CVM. For accountability, we propose that both designations report standard process metrics—numbers of designations granted, NADAs filed, and time-to-approval—for review at each ADUFA reauthorization, with the designations themselves subject to reauthorization or sunset at that time, instead of standing as permanent, unreviewed programs. We present this as a starting framework for legislative and regulatory discussion rather than as implementation-ready statutory or regulatory text, which is beyond the scope of the present analysis.

### 4.3. One Health Integration and AMR Mitigation

The relationship between veterinary drug innovation and AMR is bidirectional and strategic. The availability of novel, targeted veterinary antimicrobials reduces reliance on medically important human antibiotics. The development of effective non-antibiotic veterinary therapeutics reduces the disease burden that drives antibiotic demand. The WHO AMR Action Plan and the G7 health security agenda both identify veterinary antimicrobial use as a priority intervention domain [4]. The antimicrobial-stewardship safeguard proposed for OHID and VBDD eligibility in Section 4.2.5 is one direct mechanism for linking these policy areas in practice: conditioning designation eligibility on a stewardship and post-marketing monitoring plan ties eligibility explicitly to antimicrobial-stewardship and post-marketing monitoring requirements. Innovation policy and AMR policy are treated here as connected, not as separate tracks.

FDA’s annual antimicrobial sales reports have documented important trends. The 2023 report documented a 37% decrease in medically important antimicrobial drug sales for food-producing animals compared to the 2015 peak [35]. However, the most recent data reveal a significant reversal: the 2024 Summary Report, published in December 2025, indicated a 16% increase in sales between 2023 and 2024, representing a departure from the generally stable trend since 2017 [36]. While 2024 sales volume remains 27% below the 2015 peak, this reversal underscores a critical point: stewardship-based reductions in antimicrobial use are fragile and may not be sustainable without the availability of effective alternative therapeutic options. The 2024 increase coincided with several significant animal health challenges, including avian metapneumovirus outbreaks in US poultry and continued highly pathogenic avian influenza challenges in poultry and dairy cattle [36].

This finding is consistent with the central thesis of this review: regulatory innovation incentives and AMR stewardship are likely complementary, not competing, policy interventions. FDA itself cautions that antimicrobial sales volumes are multifactorial, reflecting changing animal populations and production practices in addition to use patterns, so the 2024 reversal cannot isolate the effect of limited therapeutic innovation; it is compatible with, but does not independently demonstrate, the hypothesis that stewardship policies alone—without parallel investment in novel veterinary therapeutics—may be insufficient to sustain long-term reductions in antimicrobial use.

### 4.4. Regulatory Preparedness for Advanced Veterinary Therapeutics

The policy reforms proposed in Section 4.2.1, Section 4.2.2, Section 4.2.3 and Section 4.2.4 address the immediate barriers to conventional veterinary drug development. However, several emerging modalities are beginning to challenge existing veterinary regulatory frameworks, which were not designed to accommodate them. Future regulatory frameworks must evolve beyond conventional small-molecule drugs to encompass at least five emerging therapeutic categories.

Gene therapy and RNA therapeutics: Adeno-associated virus (AAV)-based gene therapies, siRNA, and mRNA therapeutics are advancing rapidly in human medicine, with potential veterinary applications in hereditary conditions (e.g., mucopolysaccharidosis in cats, degenerative myelopathy in dogs) and in prophylactic and therapeutic vaccination. mRNA-based veterinary vaccines represent a particularly promising frontier: APVMA has already begun engaging with mRNA veterinary vaccine regulatory frameworks [28], but no VICH guideline currently addresses quality, safety, or efficacy requirements specific to mRNA veterinary products. A harmonized VICH guideline for nucleic acid-based veterinary products would substantially reduce the regulatory uncertainty that currently inhibits development investment.

Cell-based therapies: Mesenchymal stem cell therapies are in veterinary clinical use for orthopedic conditions, but regulatory classification varies across jurisdictions. The EU’s Regulation 2019/6 has begun to specify requirements for novel therapy VMPs including cell therapies [32], but equivalent guidance from FDA-CVM and other jurisdictions remains underdeveloped.

Microbiome therapeutics: Defined microbial consortia and engineered probiotics for livestock gut health represent an alternative to prophylactic antibiotic use with direct AMR implications. The regulatory classification of these products—as drugs, biologics, or feed additives—is inconsistent across jurisdictions, creating regulatory uncertainty.

AI-designed therapeutics and precision veterinary medicine: The application of artificial intelligence to de novo drug design, target identification, and drug repurposing has the potential to dramatically reduce development timelines and costs for veterinary therapeutics. However, regulatory frameworks for AI-designed compounds—including validation standards for AI-generated preclinical data—have not been established for either human or veterinary contexts.

The absence of regulatory frameworks for these advanced modalities threatens to replicate the innovation gap identified in this review at an even larger scale. Proactive development of adaptive regulatory pathways for advanced veterinary therapeutics—integrated into the VBDD and OHID frameworks proposed above—would position veterinary regulatory systems to accommodate the next generation of therapeutic innovation, instead of reacting to it after a decade-long lag.

### 4.5. Future Perspectives

This analysis has focused on US, EU, Japanese, and Australian regulatory systems as representing the major VICH-affiliated jurisdictions. However, other regulatory frameworks merit attention in future analyses. The Republic of Korea’s Animal and Plant Quarantine Agency (APQA) and China’s Institute of Veterinary Drug Control (IVDC) represent substantial veterinary pharmaceutical markets with distinct regulatory architectures. Comparative analysis of these systems—particularly regarding their incentive structures for novel therapeutics and their alignment with VICH harmonization—would provide a more complete global picture of the veterinary innovation landscape and is an important direction for future research.

## 5. Conclusions

The innovation gap in veterinary pharmaceuticals carries direct consequences for animal welfare, food security, zoonotic resilience, and AMR management under the One Health framework. This analysis indicates that the disparity between human and veterinary drug development pipelines is consistent with a regulatory and economic architecture that undervalues the societal returns on veterinary R&D investment, although scientific, market, and commercial factors likely also contribute and were not formally isolated in this analysis.

Two case studies—GS-441524/FIP and canine osteosarcoma—illustrate how the absence of breakthrough pathways, the practical gap between MUMS eligibility and therapeutic translation, and intellectual property barriers can translate into years-long delays and foregone translational opportunities. The multi-jurisdictional analysis indicates that this pattern is evident across the jurisdictions examined, and the VICH analysis suggests that data harmonization, while valuable, is unlikely by itself to be sufficient without parallel attention to incentive architectures.

The analysis points to four policy priorities: (i) establishment of a Veterinary Breakthrough Drug Designation (VBDD) pathway at FDA-CVM; (ii) reform of MUMS designation and creation of a One Health Innovation Designation (OHID) with enhanced exclusivity; (iii) adoption of adaptive regulatory pathways modeled on EMA PRIME; and (iv) strengthened VICH harmonization. These reforms must be designed with sufficient flexibility to accommodate the advanced veterinary therapeutics—gene therapies, RNA medicines, cell-based products, and AI-designed compounds—that will define the next generation of veterinary innovation.

Continued inaction may nevertheless carry costs through unregulated market access, challenges to antimicrobial stewardship, and preventable animal disease burden. Regulatory science plays a growing role in translating emerging veterinary therapeutics into clinical practice and in strengthening global One Health preparedness.

## Data Availability

No new experimental data were generated. The regulatory and policy information compiled for this review, including the milestone dataset underlying Figure 3, is available in and in the publicly available primary sources cited throughout the manuscript.

## Abbreviations

The following abbreviations are used in this manuscript:

ADUFA	Animal Drug User Fee Act
AMR	Antimicrobial Resistance
ANADA	Abbreviated New Animal Drug Application
APVMA	Australian Pesticides and Veterinary Medicines Authority
CNADA	Conditional New Animal Drug Application
CDER	Center for Drug Evaluation and Research
CVB	Center for Veterinary Biologics
CVM	Center for Veterinary Medicine
EMA	European Medicines Agency
CVMP	Committee for Veterinary Medicinal Products
FCoV	Feline Coronavirus
FIP	Feline Infectious Peritonitis
ICH	International Council for Harmonisation
INAD	Investigational New Animal Drug
MAFF	Ministry of Agriculture, Forestry and Fisheries (Japan)
MUMS	Minor Use/Minor Species
NADA	New Animal Drug Application
NAMs	New Approach Methodologies
NCE	New Chemical Entity
NPV	Net Present Value
ODA	Orphan Drug Act
OHID	One Health Innovation Designation
PDUFA	Prescription Drug User Fee Act
PMDA	Pharmaceuticals and Medical Devices Agency
PZAD	Priority Zoonotic Animal Drug
VBDD	Veterinary Breakthrough Drug Designation
VICH	International Cooperation on Harmonisation of Technical Requirements for Registration of Veterinary Medicinal Products
VLM	Veterinary Limited Market
VMP	Veterinary Medicinal Product
VVx	Veterinary Vaccine
